# Ensiling of total mixed ration containing persimmon peel: Evaluation of chemical composition and *in vitro* rumen fermentation profiles

**DOI:** 10.1111/asj.13403

**Published:** 2020-06-18

**Authors:** Ainissya Fitri, Taketo Obitsu, Toshihisa Sugino, Anuraga Jayanegara

**Affiliations:** ^1^ Graduate School of Biosphere Science Hiroshima University Higashihiroshima Japan; ^2^ Graduate School of Integrated Sciences for Life Hiroshima University Higashihiroshima Japan; ^3^ Department of Animal Nutrition and Feed Technology Faculty of Animal Science Bogor Agricultural University Bogor Indonesia

**Keywords:** dietary protein, fruit byproduct, tannin, TMR silage

## Abstract

The effects of inclusion of persimmon peel (PP) in total mixed ration (TMR) silage on its nutrient composition, tannin content, and in vitro ruminal fermentation were studied. Four types of TMR silages containing 0, 50, 100, and 150 g/kg of PP on a dry matter basis were prepared. The dietary contents of non‐fiber carbohydrate (NFC) decreased, while soluble protein fraction increased after ensiling of the TMR. In the TMR silages, the content of insoluble tannin increased (*p* < .05) with increasing PP level. The fraction of soluble protein decreased linearly (*p* < .01), while that of neutral detergent insoluble protein increased linearly (*p* < .01) with increasing the PP level in the TMR silages. The total gas and methane yields from the in vitro rumen fermentation of the TMR silages were lower (*p* < .01) than those of pre‐ensiled TMR and declined linearly (*p* < .01) with increasing PP level. These results indicate that adding PP to TMR silage may resist the breakdown of dietary protein during the ensiling process, although the ruminal fermentability of TMR possibly decreased after ensiling due to the loss of NFC.

## 1 INTRODUCTION

Persimmon peel (PP) is an agro‐industrial byproduct originating from the processing of dry persimmon, a popular and traditional fruit product in East Asian countries. Although the PP is usually discarded, it can be used as a feed source for animals particularly for ruminants because it contains fiber as well as some sugars (Mousa et al., 2019). The PP also contains higher level of phenolics than persimmon pulp (Gorinstein et al., 2001) and has the potential to improve the production performance and product quality of monogastric animals (Lee, Kim, & Choi, 2016; Oh, Zheng, Shin, An, & Kang, 2013). However, the fruit of some persimmon cultivars has a bitter taste caused by condensed tannin in the peel and pulp, which would hinder its consumption by animals (Taira, 1996). The high moisture and sugar contents of PP also make it susceptible to aerobic spoilage. To overcome these disadvantages, PP could be used as an ingredient of total mixed ration (TMR) silage.

Total mixed ration is used for ruminant animals as a single feed mix containing forage and concentrate diets to meet their nutrient requirements. When TMR is prepared with high‐moisture ingredients or by adding water, the quality of such fresh and high‐moisture TMR easily deteriorates aerobically after preparation, especially in a hot environment. To solve this problem, ensiling TMR could preserve it for a longer period and facilitate long‐distance transportation (Kondo et al., 2016; Nishino, Harada, & Sakaguchi, 2003; Yuan et al., 2015). Recently, adding high‐moisture agro‐industrial byproducts to TMR silages has been used in practical dairy farming (Nishino et al., 2003). TMR silage has enhanced aerobic stability after opening and can provide a nutritionally balanced feed all the year round (Nishino et al., 2003). Moreover, the TMR silage also improved the palatability of byproduct feeds (Ishida et al., 2012; Yani et al., 2015).

In addition, ensiling has been shown to enhance the utilization of ruminant feeds. Kondo, Kita, and Yokota (2004) reported that adding green tea waste to whole crop oat silage enhanced nitrogen (N) retention in goats and stabilized the protein content during the ensiling process. Ensiling with tannin‐rich feed can also produce a relatively high level of ruminal undegradable protein (Lorenz, Eriksson, & Udén, 2010; Salawu, Acamovic, Stewart, Hvelplund, & Weisbjerg, 1999) because tannin forms an insoluble complex with protein (Kondo, Hirano, Ikai, et al., 2014; Lorenz et al., 2014). Tannins can also affect ruminal fermentation, such as the ruminal ratio of acetate to propionate and methane production (Castro‐Montoya, Makkar, & Becker, 2011; Jayanegara, Togtokhbayar, Makkar, & Becker, 2009). Furthermore, persimmon tannin has a special property; the tannin in persimmon fruit is made insoluble by the air‐drying during dry fruit processing or by treatment with alcohol or carbon dioxide (Yamada et al., 2002) which are also produced in silages. Therefore, we hypothesized that during the ensiling process, the nutritional values of TMR containing PP may be altered through changing its nutrient contents and chemical properties. Thus, the objective of this study was to investigate the effects of including PP in TMR silage at different levels on its nutrient content and in vitro rumen fermentation characteristics, focusing on the soluble and insoluble fractions of dietary tannin.

## 2 MATERIALS AND METHODS

### 2.1 Silage preparation

The TMR silages were prepared using corn grain, wheat bran, soybean meal, oat hay, and alfalfa hay with or without PP. The composition of the ingredients in the TMR is shown in Table 1. Fresh PP from the fruits of Gionbou cultivar was obtained immediately after peeling from a local farmer producing dried persimmon in the middle of October at Akiota, Hiroshima, Japan, and preserved in a refrigerator at 4°C for 2 weeks until the TMR preparation. The peel contained relatively high contents of moisture (795 g/kg fresh matter) and non‐fiber carbohydrate (NFC, 544 g/kg dry matter (DM)), but a low crude protein (CP) content (48 g/kg DM, Table 1). The peel, oat hay and alfalfa hay were chopped into 2 cm lengths before preparing the TMR. Four different types of TMR containing PP at 0, 50, 100, and 150 g/kg DM basis (G0, G50, G100, and G150 treatments, respectively) were prepared to be equalized the contents of CP (160 g/kg DM) and neutral detergent fiber (NDF, 350 g/kg DM) by replacing the corn and oat hay (1:1) with PP. The moisture content of the TMR was adjusted adding water at 500 g/kg. The TMR was ensiled in triplicate polyethylene bottles (500 ml). The bottles were capped then stored in a room. The bottle silos were opened after ensiling for 180 d. The samples of pre‐ensiled TMR and TMR silages were then lyophilized and the ground (1 mm) dried samples were stored in a freezer at −20°C until analysis. Cold water extracts of the TMR silages were prepared and stored at −20°C for later analysis.

**Table 1:** Ingredients and chemical composition of total mixed ration (TMR) containing persimmon peel (PP) before ensiling

Item	PP	TMR			
		G0	G50	G100	G150
Ingredients of TMR, g/kg DM					
Oat hay		250	225	200	175
Alfalfa hay		200	200	200	200
Flaked corn		350	325	300	275
Soybean meal		100	100	100	100
Wheat bran		100	100	100	100
Persimmon peel (PP)		0	50	100	150
Nutritional compositions, g/kg DM					
Dry matter, g/kg fresh matter	205	492	467	451	451
Crude ash	43.6	46.9	47.7	45.3	49.6
Crude protein (CP)	48.4	159	166	172	162
Ether extract (EE)	16.7	30.0	27.6	27.5	31.5
Neutral detergent fiber (aNDFom)	346	337	333	306	343
Acid detergent fiber	nd	157	169	157	188
Non‐fiber carbohydrate (NFC)	544	432	430	451	417
Protein fractions, g/kg CP					
Soluble protein	nd	239	234	217	176
NDIP	nd	100.0	90.0	80.0	130.4
ADIP	nd	66.4	60.9	84.6	100.6
Tannin fractions, g/kg DM					
Soluble tannin	14.36	0.48	0.63	0.58	0.76
Insoluble tannin	13.66	0.03	0.41	1.56	1.32
Total tannin	28.02	0.51	1.03	2.14	2.09

Note

G0, TMR with no PP; G50, TMR containing PP at 50 g/kg in DM; G100, TMR containing PP at 100 g/kg in DM; G150, TMR containing PP at 150 g/kg in DM; nd, not determined; NFC = 1,000 – (crude ash + CP + EE + aNDFom); NDIP, neutral detergent insoluble protein; ADIP, acid detergent insoluble protein.

### 2.2 In vitro rumen fermentation

In vitro rumen incubation trials were conducted according to the guidelines specified by the Animal Care and Use Committee of Hiroshima University. Two rumen‐cannulated sheep were given 300 g/day corn, 65 g/day soybean meal, and 450 g/day oat hay in two equal portions at 9:00 and 17:00. The rumen fluid was taken through the rumen cannula at 4 hr after morning feeding. The rumen fluids were collected immediately from two sheep, mixed, filtered through four layers of gauze, and then transported to the laboratory within 30 min. The combined rumen fluid was mixed with a McDougall buffer solution at a ratio of 1:2 (v/v) and kept at 39°C in a water bath while being continuously flushed with CO_2_. The dried pre‐ensiled TMR and the dried TMR silages for each treatment were used as incubation substrates. The ground triplicate samples (0.3 g) were placed in a 50‐mL incubation bottle with rumen fluid‐buffer mixture (30 ml), then the bottles were incubated in a water bath at 39°C for 24 hr. The incubation was repeated twice on different days with blanks containing buffered rumen fluid. The gas production volume was measured at 24 hr after the beginning of incubation using a needle with a 100‐mL glass syringe to puncture the sealed rubber cap, and the gas was transferred into an evacuation tube for methane analysis. The pH of the incubated rumen fluid was then measured and 1.0 ml of the fluid was transferred into a centrifuge tube with 0.25 ml metaphosphoric acid. The fluid samples were stored at −20°C until the subsequent analyses.

### 2.3 Chemical analysis

The dried samples of pre‐ensiled TMR and TMR silages were analyzed for DM, crude ash, CP, and ether extract (EE) following the procedures of AOAC (AOAC, 1999). The contents of neutral detergent fiber (aNDFom, with α‐amylase and without sodium sulfite) and acid detergent fiber (ADFom) were determined according to the method of Van Soest, Robertson, and Lewis (1991). The NFC content was calculated by the difference: NFC (g/kg DM) = 1,000 – (crude ash + CP +EE + aNDFom). The protein fractions consisting of the soluble protein, neutral detergent insoluble protein (NDIP), and acid detergent insoluble protein (ADIP) in the dietary samples were analyzed as described by Licitra, Hernandez, and Van Soest (1996). The lactic acid concentration in the silage extracts was determined by an enzymatic bioassay kit (E1255; R‐Biopharm, Darmstadt, Germany) following the manufacturer's instructions. The concentrations of volatile fatty acid (VFA) in the silage extracts and in vitro rumen fluids were measured by gas chromatography (GC 17A, Shimadzu, Kyoto, Japan) equipped with a CP‐FFAP CB column (25 m × 0.32 mm ID column, Agilent, Santa Clara, CA, USA). The methane concentration in the gas samples was determined by gas chromatography (GC 7A, Shimadzu) with a stainless column (2 m × 3 mm ID) packed with a molecular sieve (5A, 60‐80 mesh, Shinwa Chemical Industries Ltd., Kyoto, Japan). The ammonia nitrogen (NH_3_‐N) concentrations of the silage extracts and in vitro rumen fluids were measured by the colorimetric method described by Okuda, Fujii, and Kawashima (1965) modified to use a microplate.

In the present study, the phenolics and tannin solubilized in the aqueous‐methanol extracts were defined as soluble phenolics and soluble tannin. The phenolics and tannin in the residue from the aqueous methanol extraction, which were extractable by acid‐methanol solution, were defined as the insoluble phenolics and insoluble tannin (Taira, 1996). Initially, the soluble phenolics were extracted twice at room temperature by homogenizing 0.5 g of the dry sample with 25 ml of 80% methanol. The soluble tannin concentration in the extracted solution was determined by a modified Folin‐Ciocalteu method using polyvinyl‐polypyrrolidone (PVPP) to separate the tannin phenolics from the non‐tannin phenolics as described by Makkar, Blümmel, Borowy, and Becker (1993). The contents of the soluble tannin were determined using tannic acid as a standard and expressed as tannic acid equivalents. Following the aqueous methanol extraction described above, the insoluble phenolics in the residue were extracted with 25 ml of 1% HCl in methanol at 60°C for 30 min. The insoluble tannin in the extracting solution was determined by a similar method (Folin‐Ciocalteu method with PVPP) of the soluble tannin analysis after neutralization with 2.5 mol/L NaOH. The total tannin contents were calculated as the sum of the soluble and insoluble fractions of tannin.

### 2.4 Statistical analyses

All statistical analyses were performed using SAS software, version 9.4 (SAS, 2015). Significance was declared at *p* < .05, and a tendency at *p* < .10. The fermentation characteristics and chemical composition of the TMR silages were analyzed using the GLM procedure, and the effect of the PP levels was further tested using orthogonal polynomial contrasts. Data from the in vitro rumen fermentation were analyzed using the MIXED procedure with a factorial design, taking into account incubation run as a random effect, with ensiling (pre‐ensiled TMR and TMR silage), PP level, and the ensiling × PP level interaction as fixed effects.

## 3 RESULTS

### 3.1 Chemical composition of pre‐ensiled TMR

The contents of DM, crude ash, EE, and CP in pre‐ensiled TMR had similar values at different PP levels (Table 1). The NDIP and ADIP contents in pre‐ensiled G150 were approximately 30% and 50% higher than those in pre‐ensiled G0, respectively. The soluble protein content in pre‐ensiled G150 was 36% lower than that in pre‐ensiled G0. The soluble tannin content was similar for all the pre‐ensiled TMR treatments. The insoluble tannin content in the pre‐ensiled TMR ranged from 0.03 to 1.6 g/kg DM.

### 3.2 Chemical composition and fermentation characteristics of TMR silages

The pH and the content of ethanol and acetic acid in the TMR silages increased linearly (*p* < .05), while the lactic acid content decreased linearly (*p* < .01) with increasing PP level (Table 2). The contents of NH_3_‐N, propionic acid, and butyric acid in the TMR silages were not affected by the PP level.

**Table 2:** Fermentation quality of total mixed ration (TMR) silages containing persimmon peel (PP) at different levels

Item	G0	G50	G100	G150	*SEM*	*P*	
						L	Q
pH	4.04	4.21	4.39	4.34	0.053	*	ns
Lactic acid, g/kg DM	10.5	8.6	7.7	7.6	0.41	**	^+^
Ammonia‐*N*, g/kg *N*	9.1	9.8	9.4	12.3	0.89	ns	ns
Ethanol, g/kg DM	11.1	11.5	10.9	19.9	1.55	*	ns
Acetic acid, g/kg DM	6.3	6.4	7.2	10.3	0.66	*	ns
Propionic acid, g/kg DM	0.0	0.4	0.3	0.4	0.08	ns	ns
Butyric acid, g/kg DM	0.1	0.1	0.1	0.0	0.02	ns	ns

Note

G0, TMR with no PP; G50, TMR containing PP at 50 g/kg in DM; G100, TMR containing PP at 100 g/kg in DM; G150, TMR containing PP at 150 g/kg in DM.

Abbreviations: L, linear effects; ns, not significant; Q, quadratic effects; *SEM*, standard error of the mean.

* *p* < .05.

** *p* < .01.

^+^ 0.05 < *p* < .10.

The CP, EE, and ADF contents in the TMR silages were not affected by the PP level, whereas the DM content decreased linearly (*p* < .01) with increasing PP level (Table 3). The crude ash and NDF contents in the TMR silages tended to increase linearly (*p* < .10) with increasing PP level. The proportion of soluble protein content in the CP of TMR silages decreased linearly (*p* < .01), while the NDIP and ADIP proportions increased linearly (*p* < .01) with increasing PP level.

**Table 3:** Chemical composition and fermentation quality of total mixed ration (TMR) silages containing persimmon peel (PP) at different levels

Item	G0	G50	G100	G150	*SEM*	*P*	
						L	Q
Chemical composition, g/kg DM							
Dry matter, g/kg fresh matter	504	485	477	471	4.7	**	ns
Crude ash	51.5	55.1	55.0	56.0	0.78	^+^	ns
Crude protein	162	167	161	173	2.1	ns	ns
Ether extract	38.2	40.3	35.5	37.2	1.09	ns	ns
Neutral detergent fiber	340	381	378	375	6.7	^+^	^+^
Acid detergent fiber	202	225	219	219	5.6	ns	ns
Non‐fiber carbohydrate	403	355	363	362	7.9	^+^	ns
Protein fractions, g/kg CP							
Soluble protein	488	483	440	397	12.7	**	ns
NDIP	109	123	142	162	6.5	**	ns
ADIP	35.0	47.7	62.8	73.7	4.65	**	ns
Tannin fractions, g/kg DM							
Soluble tannin	4.77	4.14	3.72	3.82	0.091	**	ns
Insoluble tannin	1.24	1.75	1.45	2.16	0.091	*	ns
Total tannin	6.01	5.90	5.17	5.98	0.112	ns	ns

Note

G0, TMR with no PP; G50, TMR containing PP at 50 g/kg in DM; G100, TMR containing PP at 100 g/kg in DM; G150, TMR containing PP at 150 g/kg in DM.

Abbreviations: ADIP, acid detergent insoluble protein; L, linear effects; NDIP, neutral detergent insoluble protein; ns, not significant; Q, quadratic effects; *SEM*, standard error of the mean.

* *p* < .05.

** *p* < .01.

^+^ 0.05 < *p* < .10.

The total tannin content in TMR silages was not affected by PP level. However, the soluble tannin content decreased linearly (*p* < .01), and the insoluble tannin content increased linearly (*p* < .05) with increasing PP level. The total tannin content in the TMR silages was higher than those in the pre‐ensiled TMR due to the higher soluble tannin content in TMR silages compared with that in pre‐ensiled TMR.

### 3.3 In vitro rumen fermentation

The pH in the in vitro rumen fluid was higher (*p* < .01) for the TMR silages than that for pre‐ensiled TMR and it increased linearly (*p* < .01) with increasing PP level (Table 4). The total gas and methane gas yield (mL/g DM of TMR) by in vitro rumen fermentation was lower (*p* < .01) for the TMR silages than those for the pre‐ensiled silages, and it decreased linearly (*p* < .01) with increasing PP level. There was a tendency for an interaction effect (*p* < .10) between ensiling and PP level on methane yield. The methane yield for pre‐ensiled TMR decreased with increasing PP level, while ensiled TMR produced a stable methane yield except for G100 TMR silage. Neither ammonia‐N nor total VFA concentration in the in vitro rumen fluid were affected by ensiling and PP level. However, the proportion of propionic acid was lower and the acetic‐to‐propionic acid ratio was higher for TMR silages than those for pre‐ensiled TMR. The proportion of propionic acid in the in vitro rumen fluid for pre‐ensiled TMR increased with PP level, while the reverse was observed for TMR silages (interaction effect; *p* < .05).

**Table 4:** Parameters of in vitro rumen fermentation of pre‐ensiling TMR and TMR silages containing persimmon peel (PP)

Item	Pre‐ensiled TMR				TMR silage				*SEM*	*P‐value*				
	G0	G50	G100	G150	G0	G50	G100	G150		T	P	T*P	L	Q
pH	6.22	6.26	6.27	6.28	6.34	6.36	6.37	6.37	0.009	**	**	ns	**	ns
NH_3_‐*N*, mg/dL	7.01	6.77	7.53	6.97	6.77	7.37	6.39	6.58	0.178	ns	ns	ns	ns	ns
Gas production														
Total gas yield, mL/g DM	200	194	181	188	161	156	147	158	3.0	**	**	ns	**	**
Methane yield, mL/g DM	22.1	21.6	19.0	19.8	17.1	17.1	15.6	17.0	0.35	**	**	^+^	**	*
Total VFA, mmol/L	61.4	62.0	63.0	59.9	62.2	56.7	60.9	63.5	1.47	ns	ns	ns	ns	ns
VFA composition, mol/100 mol														
Acetic acid	55.1	55.6	55.3	54.5	54.9	54.5	54.8	57.9	0.35	ns	ns	ns	ns	ns
Propionic acid	24.8	24.6	25.0	25.2	24.6	24.6	24.2	23.6	0.22	**	ns	*	ns	ns
*n*‐Butyric acid	15.2	14.8	14.9	15.5	15.6	15.4	14.4	14.1	0.23	ns	ns	ns	ns	ns
Isovaleric acid	3.18	3.13	3.00	3.05	2.98	3.23	3.02	3.06	0.075	ns	ns	ns	ns	ns
*n*‐valeric acid	1.73	1.85	1.77	1.75	1.87	1.88	1.68	1.31	0.056	ns	ns	ns	^+^	ns
A/P ratio	2.24	2.27	2.22	2.17	2.24	2.24	2.36	2.46	0.030	*	ns	^+^	ns	ns

Note

G0, TMR with no PP; G50, TMR containing PP at 50 g/kg in DM; G100, TMR containing PP at 100 g/kg in DM; G150, TMR containing PP at 150 g/kg in DM.

Abbreviations: A/P, Acetic acid to propionic acid; L, linear effects; ns, not significant; P, persimmon peel level effects; Q, quadratic effects; *SEM*, standard error of the mean; T*P, interaction effects between T and P; T, ensiling treatment effects.

* *p* < .05.

** *p* < .01.

^+^ 0.05 < *p* < .10.

## 4 DISCUSSION

### 4.1 Tannin and protein fractions in pre‐ensiled and ensiled TMR

Several analytical methods have been developed for quantifying the total tannin content in feeds. However, there is still some uncertainty about quantifying tannin content and predicting the relationship between tannin content and protein quality in silages (Lorenz et al., 2010). In the present study, tannin content was determined as soluble and insoluble fractions because the soluble or insoluble forms of tannin may affect its affinity for binding to protein and their nutritional effect. Dietary phenolics extracted by aqueous methanol (80%) were regarded as a soluble phenolic fraction containing soluble tannin. The insoluble tannin in the residue of aqueous methanol extraction can be extracted by heat treatment with acidified methanol (Taira, Ono, & Otsuki, 1998). The HCl‐methanol extracts were considered to contain both tannin and non‐tannin phenolics. Thus, we further treated the HCl‐methanol extracts with PVPP to precipitate the tannin phenolics, even though the original method of Taira (1996) for insoluble tannin measurement excluded this step. We found that the proportion of insoluble tannin accounted for a maximum of 50% and 63% of the insoluble phenolics fraction for pre‐ensiled TMR and TMR silages, respectively. Thus, including the step of precipitation of tannin phenolics with PVPP in the HCl‐methanol extracts could provide appropriate measurement of insoluble tannin content in diets.

The major chemical composition in PP used in the present study was similar to that reported in the previous study (Lee, Chung, & Lee, 2006), but it had lower NDF content than that reported by Mousa et al. (2019). The contents of total extractable phenolics (7.3 g/kg fresh matter, data were not shown) and soluble tannin (2.9 g/kg fresh matter) in PP used in the present study were slightly lower than those in the pulp of persimmon fruit (total phenolics: 8–19 g/kg fresh matter; soluble tannin: 5–10 g/kg fresh matter) reported in previous studies (Chen et al., 2016; Novillo, Salvador, Crisosto, & Besada, 2016; Taira et al., 1998). These differences in the contents of phenolics and tannin may be due to the variation among cultivars of persimmon (Chen et al., 2016).

Increasing the PP level increased the total tannin content in pre‐ensiled TMR because the peel contained 28 g/kg DM of total tannin. However, in TMR silage, the total tannin content was not affected by PP level. This difference was caused by the large increase in the soluble tannin content after ensiling which was presumably derived from the ingredients other than PP in TMR during the ensiling process because soluble tannin in G0 TMR also increased after ensiling. In a previous study on green tea byproduct silage (Kondo, Hirano, Kita, Jayanegara, & Yokota, 2014), a similar result of the increase in extractable tannin content after ensiling was also observed. In the present study, although the reason for the increase in tannin content after ensiling could not be explained clearly, the decomposition of large molecular‐size tannins to small‐size simple ones during ensiling process may increase the number of phenolics molecules and their hydroxy groups, which may apparently enhance tannin content measured by the Folin‐Ciocallteu method (Kondo, Hirano, Kita, et al., 2014). In addition, in the present study, the insoluble tannin content slightly increased after ensiling except for G100 TMR and increased with PP level. The formation of tannin–protein bond during ensiling process may contribute to the increase in insoluble tannin with increasing PP level. Alternatively, the change in the chemical properties of persimmon tannin during the ensiling process might be occurred and partially contribute to the increase in insoluble tannin content with increasing PP level in the TMR silages. The soluble tannin in astringent persimmon cultivars can be insolubilized by treatments with alcohol or carbon dioxide (Matsuo & Itoo, 1982; Yamada et al., 2002), which are also produced in ensiling process.

The proportion of the protein fraction is generally related to ruminal protein degradation; the NDIP and ADIP fractions of dietary CP have lower ruminal degradation rates than those of the soluble protein fraction (Licitra et al., 1996). In the present study, the soluble protein fraction in the TMR silages was twice that of the pre‐ensiled TMR. This remarkable increase in soluble protein might have been caused by proteolysis during the ensiling process (Kondo et al., 2016; Lorenz et al., 2010). The proportions of NDIP and ADIP in the TMR silages increased with increasing PP level, which might be related to the fluctuation of the fiber binding protein content or the increase in persimmon tannin content in the pre‐ensiled TMR with increasing PP level. The dietary protein bound to tannin is regarded as being included in the ADIP fraction, and the dietary tannin content affects the protein fraction in protein feed (Licitra et al., 1996). Albrecht and Muck (1991) reported a negative correlation between tannin content and non‐protein N content in legume silage. Salawu et al. (1999) also found that the soluble protein content of grass silage was reduced by adding a tannin powder. In a meta‐analysis, Jayanegara, Sujarnoko, Ridla, Kondo, and Kreuzer (2019) reported that increasing the tannin level reduced soluble nitrogen, free amino acid nitrogen, non‐protein nitrogen, and ammonia concentrations in silage. These suggest that persimmon tannin in TMR silage might have a role in resisting the increase in soluble protein fraction during the ensiling process. In the present study, the ratio of insoluble tannin to total tannin in TMR silage was negatively correlated with the proportion of soluble protein (*r* = −0.71, *p* < .01). However, there was no correlation (*r* = −0.01, *p* = .97) between total tannin content and the proportion of soluble protein. Even though ensilage treatment and PP affected the protein fraction in TMR, the proportion of ammonia‐N in total N of TMR silage remained unchanged, irrespective of PP level. This might have been caused by the low deamination activity in TMR silage.

### 4.2 In vitro rumen fermentation of pre‐ensiled and ensiled TMR

In the present study, the total gas yield was affected by the ensiling treatment and the PP level, which reflected the changes in NFC content during the ensiling process and with the PP level. In fact, the total gas production was positively correlated (*r* = 0.85, *p* < .01) with dietary NFC content as shown by the previous study (Getachew, Robinson, DePeters, & Taylor, 2004). Cao, Takahashi, Horiguchi, Yoshida, and Cai (2010) reported that the DM and NFC contents in TMR decreased while the EE and aNDFom contents increased after ensiling, which was confirmed in the present study. During the ensiling process, part of the NFC derived from the PP in the pre‐ensiled TMR might be converted into lactate, ethanol, and acetic acid by hetero‐fermentative bacteria (Guo, Undersander, & Combs, 2013) because the acetate and ethanol contents in the TMR silage increased with PP level. The tendency for the lower proportion of propionic acid and the higher acetate‐to‐propionate ratio in the in vitro ruminal fluid for TMR silages also reflected their higher NDF and lower NFC contents.

The ammonia‐N concentration in the in vitro* rumen* fluid after incubation did not reflect the difference in protein fraction between the pre‐ensiled and ensiled TMR or between the TMR silages, even though soluble protein is rapidly degraded in the rumen (Licitra et al., 1996). The ammonia concentration in the in vitro rumen fluid is determined by the balance between the rate of dietary protein degradation (release of ammonia) and the microbial uptake of ammonia. The ammonia released from the increased soluble protein fraction in TMR silage might have been taken up by the rumen microbes during the in vitro incubation.

Volatile fatty acids were produced from carbohydrates fermentation in the rumen and can be absorbed via rumen wall to supply energy substances for host animals. Generally, gas production reflects total VFA production and their composition by the in vitro rumen fermentation. However, in the present study, total VFA concentration was not affected by ensiling treatment and PP level, and the response of total VFA was not similar with that of gas production which was higher for pre‐ensiled TMR than TMR silages and decreased with increasing the PP level. Although the reason for this discrepancy was not clear, gas production is generated not only from microbial fermentation of diets but also from buffering of acids in the rumen (Getachew et al., 2004). Based on the response of VFA concentration after in vitro incubation, we presumed that the energy supply for host animals from VFA produced in the rumen would be less affected by ensiling or PP levels of the TMR.

Methane production in ruminants contributes to the greenhouse gas effect and global warming. Methane production also represents a loss in energy and therefore a decrease in feed efficiency (Eckard, Grainger, & de Klein, 2010). In previous studies, high levels of methane production, and low propionate and high acetate concentrations in the in vitro rumen were observed in TMR silage compared with non‐ensiled TMR (Chao et al., 2016; Uyeno et al., 2016). However, we observed a lower methane yield from TMR silage than from pre‐ensiled TMR agreeing with the results reported by Cao et al. (2010). The reduction in the level of fermentation substrates caused by the loss of NFC during ensiling process described earlier can be considered as a possible reason for the reduction in methane yield.

Dietary phenolics and tannins can also suppress total gas and methane production (Jayanegara et al., 2011). In the present study, the contents of soluble and total tannin increased after ensiling. The soluble tannin fractions might be more effective at suppressing microbial growth in the rumen because their lower molecular weight could allow then to bind strongly with microbial enzymes (Field, Kortekaas, & Lettinga, 1989). There was also a tendency for an interaction effect between ensiling treatment and PP level on methane yield; the inhibition of methane yield with the increasing PP level was more pronounce for pre‐ensiled TMR. This suggested that the difference in the tannin fraction (soluble or insoluble tannin) also affected ruminal methane production. Insolubilizing persimmon tannin during the ensiling process may reduce the inhibitory effect of tannin on methanogens.

In conclusion, the soluble and insoluble fractions of tannin in TMR containing PP related to its ruminal fermentation properties. Adding PP to TMR silage may resists the breakdown of dietary protein during the ensiling process, even though ensiling of TMR possibly reduces ruminal fermentability as well as methane yield due to the loss of NFC.
